# BubR1 controls starvation-induced lipolysis via IMD signaling pathway in *Drosophila*

**DOI:** 10.18632/aging.205533

**Published:** 2024-02-08

**Authors:** Mengyou Liu, Shengye Yang, Jingsi Yang, Ping Feng, Feng Luo, Qiaoqiao Zhang, Li Yang, Hao Jiang

**Affiliations:** 1 Laboratory for Aging and Cancer Research, Frontiers Science Center Disease-related Molecular Network, State Key Laboratory of Respiratory Health and Multimorbidity and National Clinical Research Center for Geriatrics, West China Hospital, Sichuan University, Chengdu, Sichuan 610041, China; 2 Clinical Trial Center, National Medical Products Administration Key Laboratory for Clinical Research and Evaluation of Innovative Drugs, West China Hospital, Sichuan University, Chengdu, Sichuan 610041, China; 3 Department of Medical Oncology, Cancer Center, West China Hospital, Sichuan University, Chengdu, Sichuan 610041, China; 4 Lung Cancer Center, West China Hospital, Sichuan University, Chengdu, Sichuan 610041, China; 5 Department of Gastroenterology and Hepatology and Sichuan University-University of Oxford Huaxi Joint Centre for Gastrointestinal Cancer, Frontiers Science Center for Disease-related Molecular Network, West China Hospital, Sichuan University, Chengdu, Sichuan 610041, China

**Keywords:** BubR1, lipolysis, metabolic adaptation, immunity and metabolism, Relish, Bmm

## Abstract

Lipolysis, the key process releasing fat acids to generate energy in adipose tissues, correlates with starvation resistance. Nevertheless, its detail mechanisms remain elusive. BubR1, an essential mitotic regulator, ensures proper chromosome alignment and segregation during mitosis, but its physiological functions are largely unknown. Here, we use *Drosophila* adult fat body, the major lipid storage organ, to study the functions of BubR1 in lipolysis. We show that both whole body- and fat body-specific BubR1 depletions increase lipid degradation and shorten the lifespan under fasting but not feeding. Relish, the conserved regulator of IMD signaling pathway, acts as the downstream target of BubR1 to control the expression level of Bmm and modulate the lipolysis upon fasting. Thus, our study reveals new functions of BubR1 in starvation-induced lipolysis and provides new insights into the molecular mechanisms of lipolysis mediated by IMD signaling pathway.

## INTRODUCTION

Lipids, one of the three major energy sources for vertebrates and invertebrates, are degraded to meet energy needs upon energy negative balances such as starvation and long-term exercise [1, 2]. In response to the energy shortage, cellular lipid reserves are mobilized to produce glycerol and free fatty acids (FFAs), followed by the catalysis and hydrolysis by a series of lipases, which is called lipolysis [3, 4]. Depending on the optimum pH value for lipases, intracellular lipolysis pathways are mainly subclassified into (a) acid lipolysis, which mediates lipid degradation in lysosome with an optimum pH value around 5, and (b) neutral lipolysis, which exerts cytosolic lipid hydrolysis with an optimum pH value around 7 [3, 5]. The acid lipolysis, termed lipophagy as well, is achieved by the sole lipase lysosomal acid lipase (LAL), whereas neutral lipolysis, the most canonical pattern of lipid mobilization, is implemented by three main lipases: adipose triglyceride lipase (ATGL), hormone-sensitive lipase (HSL) and monoacylglycerol lipase (MGL), which consecutively and separately hydrolyze triacylglycerol (TAG), diacylglycerol (DAG) and monoacylglycerol (MAG) to liberate free fatty acids (FFAs) and glycerol for energy production [3, 5]. Of note, it is ATGL that initiates the lipolytic cascade, namely triacylglycerol hydrolysis, to form diacylglycerol and FFAs [6].

The regulations of lipolysis mainly rely on the regulations of lipases including transcriptional and post-transcriptional modulations [4, 7]. In *Drosophila*, TAG hydrolase Brummer (Bmm), the homolog of human ATGL, also acts as the major executor for TAG hydrolysis and lipid degradation in fat body, which is similar to the adipose and hepatic tissues in vertebrates [8–11]. Under the nutrient deprivation, the transcription factor Forkhead box subgroup O (FOXO), the prominent downstream of insulin pathway, acts as the main regulator of Bmm to promotes its transcription [1, 7, 12]. Accordingly, activated Akh signaling pathway enhances FOXO-dependent Bmm transcription mediated by LKB1-SIK3-HDAC4 to promote lipolysis upon fasting [13, 14]. Kruppel homolog 1 (Kr-h1) represses starvation-induced TAG catabolism by antagonizing FOXO-mediated Bmm expression [15]. The IMD signaling pathways are activated by pathogen-associated molecular patterns, initiating both cellular and humoral immune processes to combat invaders [16]. Meanwhile, Imd pathways also play pivotal roles in regulating the host’s nutrient metabolism in organs such as the fat body and gut. The IMD pathway activated by the membrane-associated receptor PGRP-LC contributes to promote host metabolic homeostasis in Drosophila midguts [17]. The NF-κB family member (p100 and its homolog p105, known as Relish in *Drosophila*), a critical downstream component of the IMD signaling pathway, inhibits FOXO-mediated Bmm transcription to regulate lipolysis under conditions of nutrient abundance [18]. In Drosophila, Mef2 acts as an immune-metabolic switch, also highlighting the intricate connection between immune responses and metabolic processes [19]. Additionally, AMP, a critical immune effector molecule activated through the evolutionarily conserved IMD pathways, is regulated by the transcription factor FOXO which serves as a key regulator of lipid metabolism [20]. However, the detailed relationship between IMD signaling pathway and starvation-induced lipolysis, and its intrinsic mechanisms remain ambiguous.

BubR1 controls spindle assembly checkpoint (SAC), proper chromosome congression and alignment during mitosis [21–25]. Meanwhile, a series of studies show the physiological roles of BubR1 in development and carcinomas [26–31]. Mutant mice with low expression level of BubR1 (BubR1 hypomorphic or BubR1^H/H^ mice) perform as one of classical models of progeria and display various significant abnormalities including cachectic dwarfism, fat loss, reduced stress tolerance, impaired wound healing, lordokyphosis, sarcopenia, cataracts, craniofacial dysmorphisms, arterial stiffening and shortened lifespan [32, 33]. Additionally, BubR1 may regulate stem cell differentiation, and its promoter methylation contributes to BubR1 inactivation during the natural aging process [34, 2]. BubR1 insufficiency also results in hippocampal neurogenesis dysfunction, brain development abnormalities [35, 36], cerebral degeneration [37], impaired affective behavior [38], vascular aging and dysfunction [39–41], female infertility [32, 42], impaired liver regeneration after hepatectomy [43], aging-related hearing loss (ARHL) or presbycusis [44], and abnormal Insulin Receptor (IR) in hepatocytes [45, 46]. Nevertheless, there is no clear evidence to demonstrate its impact on lipolysis. Based on the big-scale screen for mitotic regulators implicated in lipid metabolism, in this study, we leverage adult fat body of fruit flies as a model to reveal the role of the canonical mitotic regulator BubR1 on governing starvation-induced lipolysis mediated by IMD signaling pathway and Bmm, uncovering that BubR1 works as the upstream regulator of Relish to regulate lipolysis upon fasting via the IMD signaling pathway, providing a critical link between BubR1 and energy metabolism.

## RESULTS

### BubR1 inhibits lipid degradation under starvation

**Figure 1:**
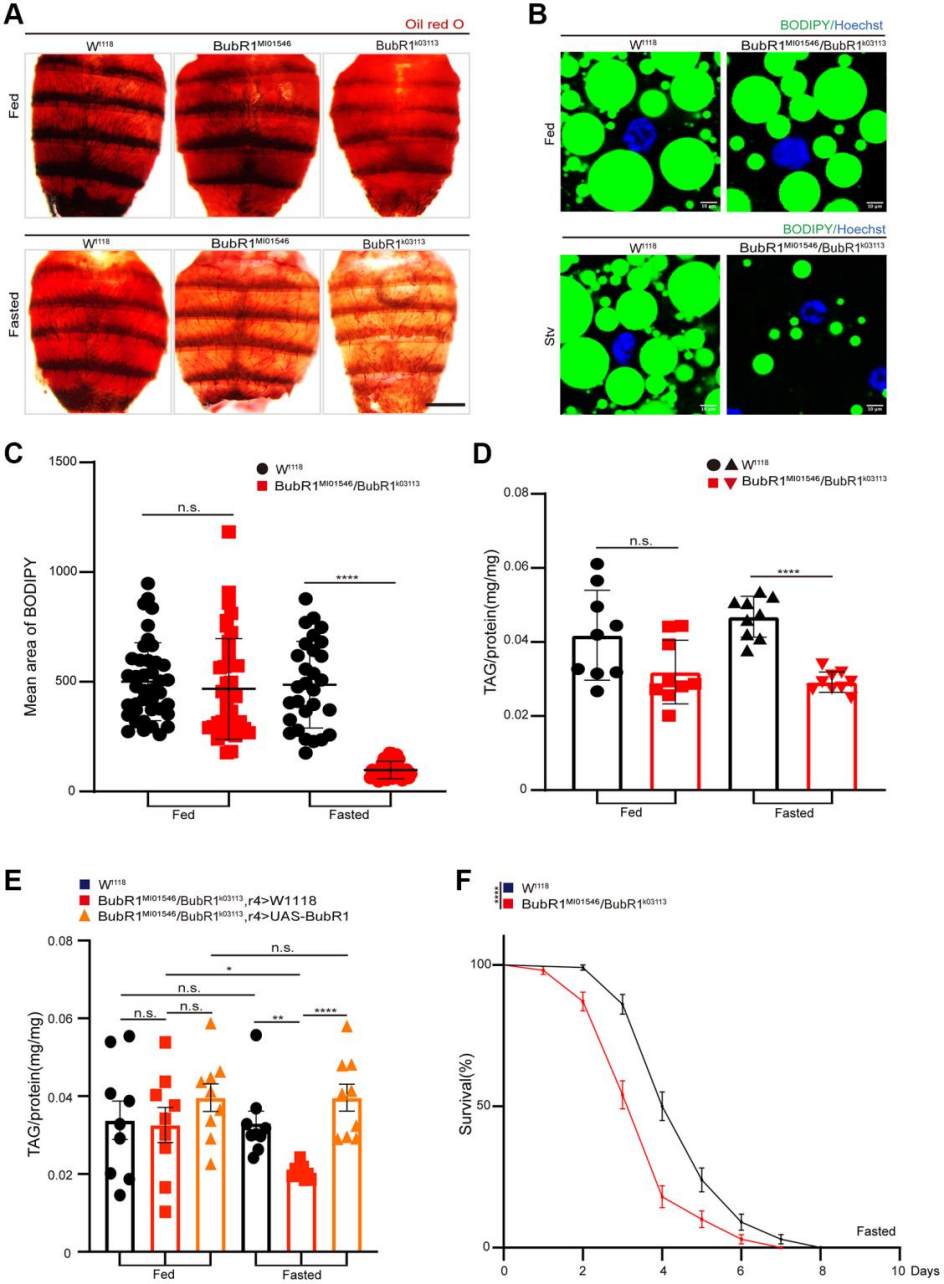
**BubR1 inhibits lipid metabolism degradation under starvation.** (**A**) Representative images of Oil red O (ORO) stain of dissected fat bodies from W^1118^, BubR1^MI01546^, BubR1^k03113^ before and after starvation (72 h). (**B**) Bodipy stain of dissected fat body of W^1118^, BubR1^MI01546^/BubR1^k03113^ before and after starvation (72 h). Bodipy (neutral lipids; green) and Hoechst (Hoechst; blue) detected through fluorescent histochemistry. (**C**) The dot graph of the mean area of lipid droplets among the more than 30 ROI (region of interest) from W^1118^, BubR1^MI01546^/BubR1^k03113^ before and after starvation (72 h). Each dot corresponds to one ROI. (**D**) Quantification of total triglyceride (TAG) levels of whole flies in control and BubR1^MI01546^/BubR1^k03113^ before and after starvation (72 h). *n* = 9 samples. (**E**) The total triglyceride (TAG) levels of female flies (W^1118^, BubR1^MI01546^/BubR1^k03113^; r4-Gal4> W^1118^ and BubR1^MI01546^/BubR1^k03113^; r4-Gal4> UAS-BubR1) before and after starvation (72 h), *n* = 9 samples. (**F**) Starvation resistance of female flies in W^1118^, BubR1^MI01546^/BubR1^k03113^. *n* = 4 cohorts (total 80 flies). Data are presented as percents and SE. Scale bars represent 1000 μm (**A**), 10 μm (**B**). Without notification, Data are presented as mean and SD. Student’s *t*-tests were performed. ^*^p < 0.05, ^**^p < 0.01, ^***^p < 0.001, ^****^p < 0.0001, and Abbreviation: n.s.: non-significant represents *p* > 0.05.

To address whether BubR1 regulates energy metabolism *in vivo*, we used two BubR1-deficient strains of *Drosophila* BubR1^MI01546^ and BubR1^k03113^ (Supplementary Figure 1A) to study the functions of BubR1 in carcass fat body, which stored the lipids to generate energy and acted as a crucial sensor linking nutrient status to energy metabolism [47–52]. Lipid mobilization, the process releasing fatty acids from fat stores to generate energy, usually occurred during metabolic stresses like starvation [2]. Accordingly, we found BubR1^MI01546^ and BubR1^k03113^ flies showed the reduced storage of neutral lipids under starvation but not under normal feeding in *Drosophila* fat bodies stained with Oil red O or Bodipy (Figure 1A–1C). Additionally, different from wildtype flies exhibiting no change of TAG level under nutrient deprivation, BubR1^MI01546^/BubR1^k03113^ heterozygous flies displayed accelerated decrease of TAG level during acute starvation, which was rescued by BubR1 overexpression in the fat body (r4-Gal4>UAS-BubR1) (Figure 1D, 1E). Since previous studies revealed that energy metabolism in *Drosophila* affected the individual lifespan [53–55], we consequently found that BubR1^MI01546^/BubR1^k03113^ heterozygous flies displayed shortened lifespan compared with controls upon fasting (Figure 1F).

To further confirm the effects of BubR1 on lipid degradation under metabolic adaption, we inhibited BubR1 expression by using two independent RNAi lines (BubR1 RNAi #1 and BubR1 RNAi #2) via *Act5C-GAL4, tub-Gal80^TS^* driver (Act5C^TS^). With achieving a significant knockdown of BubR1 mRNA level (Supplementary Figure 1B), BubR1 RNAi in the whole body diminished the level of TAG and neutral lipid storage in adult fat bodies upon fasting, but not feeding (Supplementary Figure 1C–1F). Moreover, we found that BubR1 RNAi in larvae accelerated the loss of TAG storage upon fasting, but not feeding (Supplementary Figure 1G). Furthermore, depletion of BubR1 (Supplementary Figure 1H, 1I) in starved Hela and HepG2 cells also suppressed lipolysis, as indicated by the reduced size of BODIPY-stained lipid droplets (Supplementary Figure 1J–1L) and the decrease in TAG levels (Supplementary Figure 1M, 1N) and. In addition, BubR1 depletion in adults decreased the lifespan under starvation (Supplementary Figure 1O). Thus, BubR1 attenuated lipid catabolism throughout the course of metabolic adaptation.

### BubR1 functions autonomously in Drosophila fat body to control lipid metabolism upon fasting

**Figure 2:**
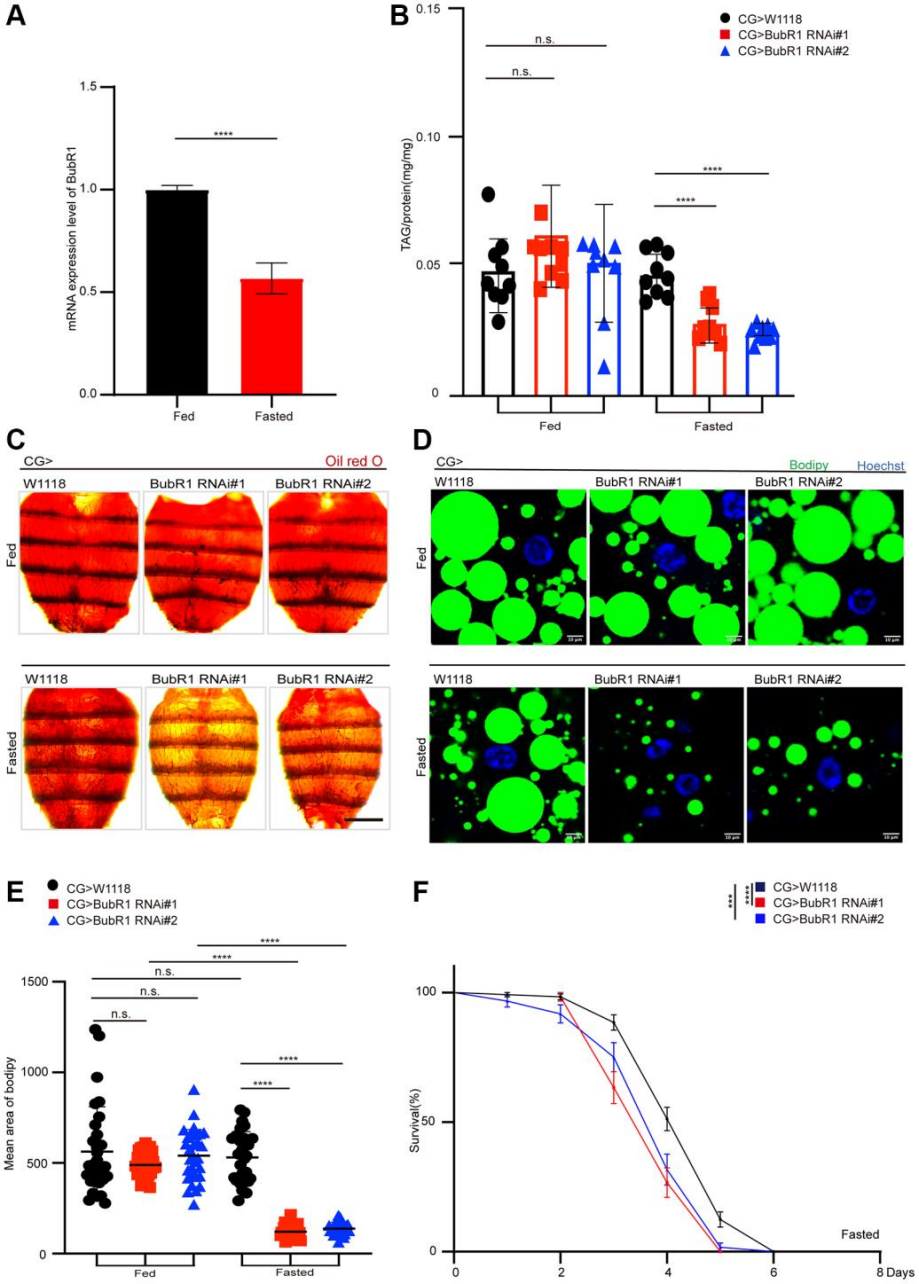
**BubR1 functions autonomously in *Drosophila* fat body to control lipid metabolism upon fasting.** (**A**) The mRNA expression level of BubR1 in the fat body of wildtype before and after starvation (72 h). The ratio of each band indicates the relative amount of BubR1 normalized by rp49 expression. Results are representative of three biological repetitions. (**B**) The total triglyceride (TAG) levels of flies before and after starvation (72 h) with specially expressing W^1118^, BubR1 RNAi#1 and BubR1 RNAi#2 in the fat body driven by CG-GAL4. *n* = 9 samples. (**C**) Representative images of Oil red O (ORO) stain of dissected fat bodies from female flies before and after starvation (72 h) with specially expressing W^1118^, BubR1 RNAi#1 and BubR1 RNAi#2 in the fat body driven by CG-GAL4. (**D**) Bodipy stain of dissected carcass/fat body of flies before and after starvation (72 h) with specially expressing W^1118^, BubR1 RNAi#1 and BubR1 RNAi#2 in the fat body driven by CG-GAL4. Bodipy (neutral lipids; green) and Hoechst (Hoechst; blue) detected by fluorescent histochemistry. (**E**) Quantification of the mean area of lipid droplets among the more than 30 ROI (region of interest) from flies before and after starvation (72 h) with specially expressing W^1118^, BubR1 RNAi#1 and BubR1 RNAi#2 in the fat body driven by CG-GAL4. Each dot corresponds to one ROI. (**F**) Starvation resistance of female flies with specially expressing W^1118^, BubR1 RNAi#1 and BubR1 RNAi#2 in the fat body driven by CG-GAL4. *n* = 4 cohorts (total 80 flies). Data are presented as percents and SE. Scale bars represent 1000 μm (**C**), 10 μm (**D**). Without noted, Data are presented as mean and SD. Student’s *t*-tests are performed. ^*^p < 0.05, ^**^p < 0.01, ^***^p < 0.001, ^****^p < 0.0001, and Abbreviation: n.s.: non-significant represents *p* > 0.05.

To further investigate BubR1 autonomous functions in fat bodies under the dietary restriction, we firstly tested the mRNA expression level of BubR1 in fat bodies of flies treated with either feeding or fasting. We found that the flies treated with fasting displayed a decrease of BubR1 mRNA level in the fat bodies compared with the flies treated with feeding (Figure 2A). In addition, the flies with specifically depleting BubR1 in the fat body driven by *CG-GAL4* showed a starvation-induced decrease of lipid storage and shortened lifespan (Figure 2B–2F). As expected, similar fasting-induced BubR1 RNAi phenotypes on lipid degradation and survival rate were observed with an independent fat body driver (*r4-GAL4*) (Supplementary Figure 2A–2E). Taken together, our data validated that BubR1 suppressed lipid degradation autonomously in the fat body during metabolic adaptation.

### BubR1 regulates lipid degradation via lipolysis mediated by IMD signaling pathway during acute starvation

**Figure 3:**
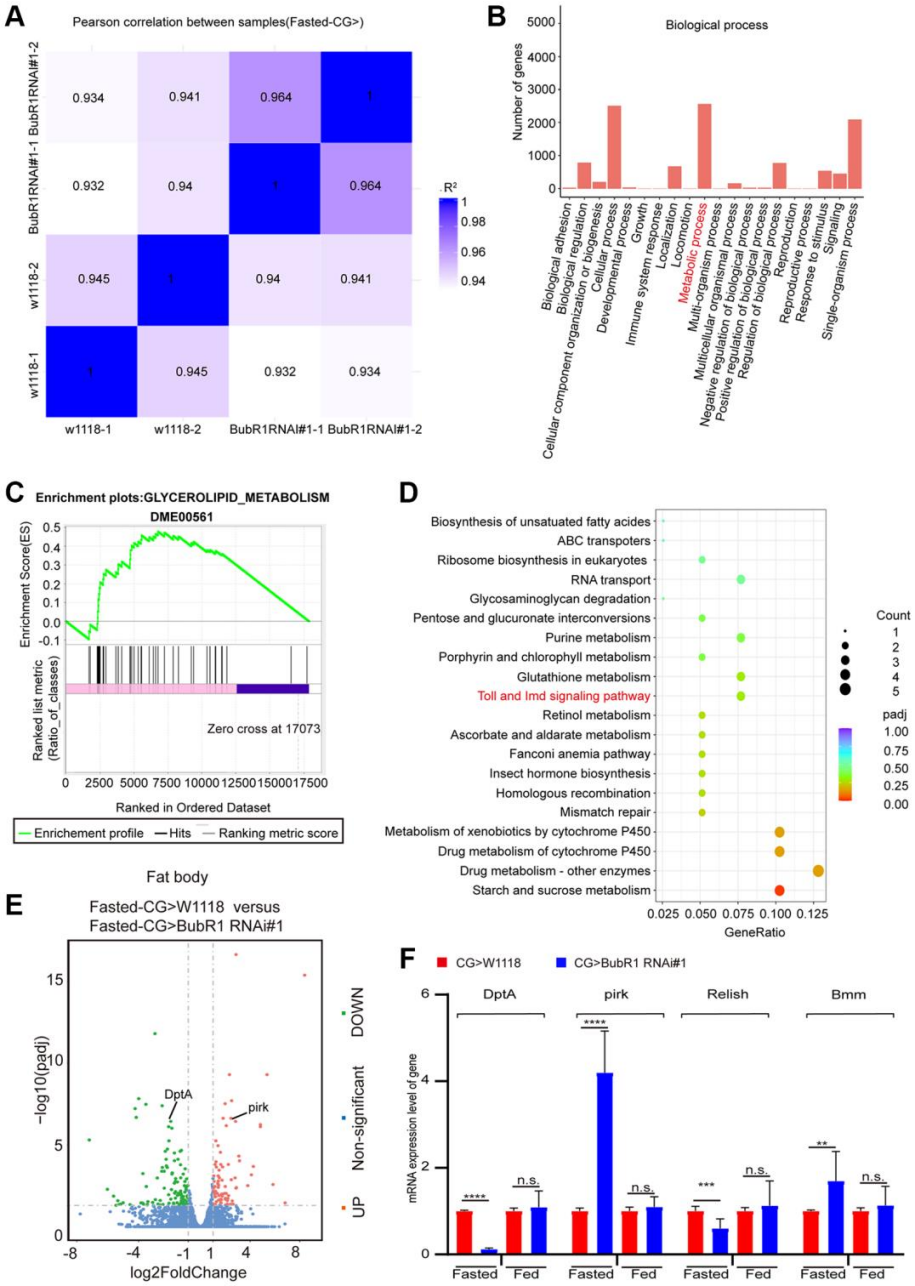
**BubR1 regulates lipid degradation via lipolysis mediated by IMD signaling pathway during acute starvation.** (**A**) The Pearson correlation analysis between samples. (**B**) GO classification analysis of enriched genes in biological process in a pair-wise comparison of control to fat body-deficient BubR1 flies under starvation. (**C**) GSEA plots of ranked gene expression comparing fat body-deficient BubR1 with wild type and their positive (pink) and negative (purple) correlations for the indicated gene sets. Enrichment scores are represented as green lines, and the horizontal black bars indicate the position of the associated genes for each enrichment set. ES = 0.48, |NES| = 1.13, *p* < 0.05. (**D**) KEGG pathway enrichment analysis. KEGG pathway enrichment of up- or downregulated genes comparing fat body-deficient BubR1 and wild type under fast starvation. Both adjusted *p*-value and gene ratio denote the significance of the pathway. (**E**) Volcano plots of differentially expressed genes in a pair-wise comparison of control to fat body-deficient BubR1 flies in condition of fast starvation. (**F**) Relative mRNA levels of DptA, pirk, Relish and Bmm in flies with expressing W^1118^, BubR1 RNAi#1 and BubR1 RNAi#2 in the fat body driven by CG-GAL4 before or after starvation. The *p*-values were calculated from respective control by an unpaired Student’s *t*-test. Results are representative of three biological repetitions (mean ± SD). Student’s *t*-tests are performed. ^*^p < 0.05, ^**^p < 0.01, ^***^p < 0.001, ^****^p < 0.0001.

To understand how BubR1 governs lipid metabolism, we next performed RNA-sequencing (RNA-seq) by dissecting fat bodies from wildtype or BubR1 RNAi #1 flies treated with starvation. With the correlation analysis between samples was performed (Figure 3A), our RNA-seq data of analyzing Gene Ontology (GO) classification revealed that over 2000 genes were enriched in metabolic process, implying the role of BubR1 on metabolism (Figure 3B). Further Gene Set Enrichment Analysis (GSEA) displayed positive correlations between BubR1 depletion and glycerolipid metabolism (Figure 3C). Furthermore, we found that IMD signaling pathway, the main immunity pathway responsible for Gram- bacterial infection and lipolysis [16, 56, 57], showed the substantially decreased level in BubR1-insufficient fat bodies, analyzed by Kyoto Encyclopedia of Genes and Genomes (KEGG) pathway enrichment analysis (Figure 3D). The antibacterial peptide (AMP) DptA decreased, whereas pirk increased in BubR1-deficient flies upon fasting, which were both implicated in the IMD signaling pathway (Figure 3E). Since both DptA and pirk worked as the downstream targets of Relish, the key regulator that modulated starvation-induced lipolysis via antagonizing FOXO-mediated Bmm transcription in the IMD signaling pathway, we examined the expression levels of pirk, DptA, Relish and Bmm by performing RT-qPCR in either whole body- or fat body-specific BubR1 deficient flies treated with fasting. Accordingly, under starvation but not feeding, the mRNA levels of Bmm and pirk were augmented, whereas the levels of Relish and DptA were diminished (Figure 3F and Supplementary Figure 3A, 3B). Interestingly, despite previous study indicated Relish increased the pirk expression level during Gram-negative bacterial infection [58], our result showed Relish overexpression in *Drosophila* fat bodies decreased pirk expression under starvation (Supplementary Figure 3C), which might explain why BubR1 deficiency suppressed Relish mRNA level and increased pirk expression upon fasting at the same time. To further explore the mechanism of BubR1 in regulating Relish expression, we performed luciferase assay to study whether BubR1 controlled the transcription of Relish, unravelling that Flag-BubR1 overexpression increased the transcription of Relish compared with Flag-EGFP (Supplementary Figure 3D). Additionally, to further investigate whether BubR1 regulates the protein level of Relish, we examine the impact of BubR1 in both the protein and ubiquitination levels of Relish under starvation in *Drosophila* adult fat body. The result indicated that BubR1 did not affect the protein stability of Relish upon fasting (Supplementary Figure 3E, 3F). Collectively, we implied that BubR1 RNAi might accelerate lipiolysis by affecting the levels of Relish and Bmm through the IMD signaling pathway upon fasting.

### BubR1 suppresses lipolysis by inhibiting relish-mediated Bmm expression upon fasting

**Figure 4:**
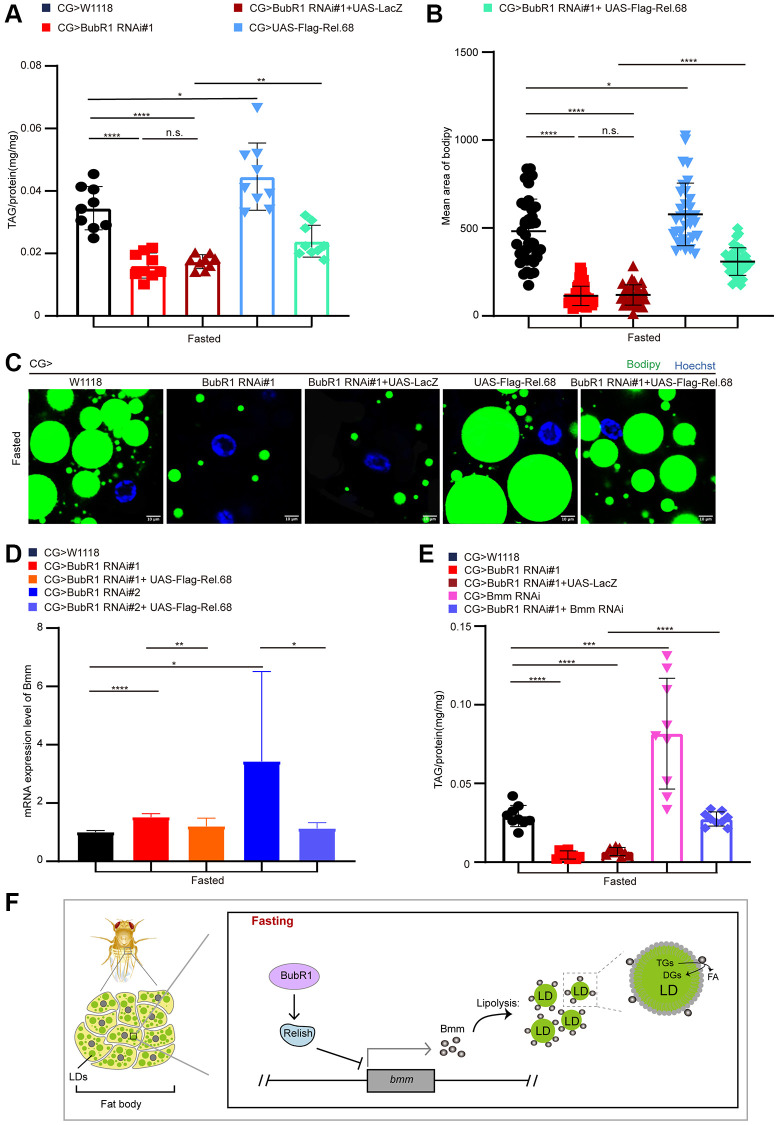
**BubR1 suppresses lipolysis by inhibiting Relish-mediated Bmm expression upon fasting.** (**A**) The TAG level of flies with specially expressing W^1118^, BubR1 RNAi#1, BubR1 RNAi#1+UAS-LacZ, UAS-Flag-Rel.68, and expressing BubR1 RNAi#1 with UAS-Flag-Rel.68 in the fat body driven by CG-GAL4 under starvation. *n* = 9 samples. (**B**) Quantification of the mean area of lipid droplets among the more than 30 ROI (region of interest) in flies under starvation with expressing W^1118^, BubR1 RNAi#1, UAS-Flag-Rel.68, and expressing BubR1 RNAi#1 with UAS-Flag-Rel.68 in the fat body. Each dot corresponds to one ROI. (**C**) Bodipy staining of flies with expressing W^1118^, BubR1 RNAi, UAS-Flag-Rel.68, and expressing BubR1 RNAi with UAS-Flag-Rel.68 in the fat body. Bodipy (neutral lipids; green) and Hoechst (Hoechst; blue) detected by fluorescent histochemistry. (**D**) The Bmm mRNA level of flies with expressing W^1118^, BubR1 RNAi, and expressing BubR1 RNAi with UAS-Flag-Rel.68 in the fat body via CG-GAL4. Results are representative of three biological repetitions. (**E**) The TAG level of flies with specially expressing W^1118^, BubR1 RNAi#1, BubR1 RNAi#1+UAS-LacZ and expressing BubR1 RNAi#1 with Bmm RNAi in the fat body by CG-GAL4. *n* = 9 samples. (**F**) Model of BubR1 regulating lipid metabolism under starvation. Scale bars represent 10 μm (**C**). Without noted, Data are presented as mean and SD. Student’s *t*-tests are performed. ^*^p < 0.05, ^**^p < 0.01, ^***^p < 0.001, ^****^p < 0.0001, and Abbreviation: n.s.: non-significant represents *p* > 0.05.

To confirm whether BubR1 controls the starvation-induced lipolysis via Relish, we overexpressed Relish in wildtype or BubR1-depleted fat body using CG-Gal4. It showed Relish overexpression increased TAG level in wildtype fat body and reversed the decreased TAG level in BubR1 deficient fat body upon fasting (Figure 4A and Supplementary Figure 4A). Consistently, overexpressing Relish amplified the size of LDs and rescued the accelerated LD consumption caused by BubR1 RNAi (Figure 4B, 4C and Supplementary Figure 4B, 4C). As Relish was reported to diminish the lipolysis to maintain lipid homeostasis by impairing FOXO-mediated Bmm transcription under nutrient deprivation, we further studied whether Bmm, the prominent lipase of lipolysis, acted as the downstream of BubR1 to mediate the lipolysis upon nutrient expropriate. As expected, BubR1 RNAi increased Bmm mRNA level upon fasting, which was reversed by overexpressing Relish (Figure 4D). Besides, Bmm depletion recovered the TAG shortage in BubR1 RNAi flies under starvation (Figure 4E and Supplementary Figure 4D). Furthermore, the expression level changes of both pirk and DptA were reversed by Relish overexpression in BubR1 depleted flies (Supplementary Figure 4E). Therefore, BubR1 governed Relish to regulate Bmm expression and Bmm-mediated lipolysis under nutrient deprivation (Figure 4F).

## DISCUSSION

*Drosophila* fat body, the major metabolic organ, is crucial for individual survival upon energy withdrawal. Here, we use adult fat body to demonstrate that BubR1, one of the crucial mitotic guardians, performs its functions to diminish lipolysis and prolong individual lifespan upon fasting. Since previous studies have proved that p31^comet^-MAD2-BubR1 mitotic regulatory network plays a role in glycogen metabolism [45, 46], here our study also reveals a new function of BubR1 in starvation-induced lipolysis *in vivo*, which also proposes that other key mitotic regulators may be involved in energy metabolism. Accordingly, based on this foregoing hypothesis and our large-scale screening for mitotic regulators implicated in lipid metabolism, we also find that other key SAC effectors monitoring the chromosome alignment, control the lipid degradation (unpublished data). Our findings open an avenue to explore physiological functions of mitotic regulators in energy metabolism and decipher a mechanism of lipid metabolism beyond the canonical regulators and pathways.

In this study, we manifest that BubR1 mediates the IMD signaling pathway to regulate fasting-induced lipolysis by affecting the level of Relish, uncovering a new upstream regulator of Relish to modulate metabolic adaption upon nutrient withdrawal. It also implies that BubR1 may function in immunity-associated lipid metabolism, supporting that individual immunity plays a role on its metabolic homeostasis. Additionally, it will be important to investigate how BubR1 adapts to the regulation of Relish-mediated IMD signaling pathway. Despite the C-terminus of BubR1 contains a catalytic serine/threonine kinase domain implementing the functions of the whole protein in different physiological processes, BubR1 kinase activity is not required for some specific biological processes [25, 59–62]. Hence, BubR1 may function in starvation-induced lipolysis through the IMD signaling pathway in a manner of kinase- dependent or independent way. On the other hand, the key regulator Relish in the IMD signaling cascade, is phosphorylated by IkB kinase (IKK) complex that is also needed to be phosphorylated and activated by Tak1 to exert its transcription effects [16, 63, 64]. If BubR1 relies on its C-terminal kinase activity to control the IMD-mediated lipolysis upon fasting, it may directly phosphorylate Relish or IKK to activate Relish and IMD signaling pathway, which will be interesting topics to address in the future. Moreover, our data shows BubR1 depletion decreases the expression level of the antibacterial peptide DptA, the downstream target of Relish in the IMD signaling pathway. Since the mild downregulation of antibacterial peptides of IMD signaling pathway increase fat content, stress resistance and lifespan in *Drosophila* [65], inferring that DptA may regulate lipid metabolism under nutrient deprivation directly. Previous reports indicate that SIRT2 maintains lysine-668 of BubR1 in a deacetylated state to increase life span in mice [66]. Moreover, Cdk1, Plk1, Mps1 and Aurora B contribute to the highly-phosphorylated BubR1 [25]. In addition, according to our previous study, Bub1/Bub3 regulate metabolic adaptation via macrolipophagy [67]. Besides, Bub1, Bub3 and BubR1 are reported to act as a complex during mitotic regulation [68, 69]. Thus, the regulators that modulate the epigenetic modification of BubR1 including SIRT2, Cdk1, Plk1, Mps1 and Aurora B, and its binding adaptors such as Bub1 and Bub3, might contribute to the specific function of BubR1 during fasting conditions, which deserve further exploration and elucidation.

Beyond its role on the lipolysis, the IMD signaling pathway is also involved in anti-bacterial infection [56, 70, 71], somatic sex determination [72], homeostatic synaptic plasticity [73], intestinal epithelial turnover [70], elimination of unfit tissue cells [74, 75], sleep [76, 77], neurodegeneration [78–80], female oviposition [81], lifespan regulation [82–85] and tumorigenesis [86]. Since BubR1 attenuates the fasting-induced lipolysis through the IMD signaling pathway, it will be interesting to uncover whether BubR1 play roles on forementioned biological processes via the IMD signaling pathway, and whether BubR1 exerts its effects on other IMD-mediated physiological activities through the key downstream target Relish.

## MATERIALS AND METHODS

### Drosophila stocks and husbandry

All flies were raised at 25°C and 65% humidity with a 12 h light/dark cycle, feeding with standard medium (cornmeal 50 g, yeast 18.75 g, sucrose 80 g, glucose 20 g, agar 5 g, and propionic acid 30 mL in 1L water). All crosses driven by non-temperature sensitive GAL4 carried out at room temperature, and W^1118^ flies were used as a control in all experiments. Cross of gene overexpression or knockdown mediated by temperature sensitive GAL4 was performed at 18°C, to induce transgene expression, first-instar larvae were subjected to 37°C water bath for a 2-hour heat shock, and then maintained at 29°C for 3 days, for adults, after eclosion, transferred the progeny into 29°C for 7 days. Except special explanation, 7-day-old mated females were utilized in all experiments. Drosophila lines mentioned in this study are listed as follows: Act5C-GAL4/CyO; tub-gal80ts/TM6B (named Act5C^TS^), CG-Gal4 (w(1118); P(w(+mC) = Cg-GAL4.A)2, BDSC#7011), r4-Gal4 (y(1) w(×); P(w(+mC) = r4-GAL4)3, BDSC #33832), W^1118^ (BDSC#3605); BubR1 mutant allele BubR1^MI01546^ (Mi(MIC)BubR1^MI01546^, BDSC# 34208) and BubR1^k03113^ (P(lacW)BubR1^k03113^, BDSC# 10526). UAS-dsRNA lines were as follows: BubR1RNAi (P (TRiP.GL00236) attP2, BDSC# 35329, named #1 in text), BubR1 RNAi (P(TRiP.GLV21065) attP2, BDSC# 35700, named #2 in text), Bmm RNAi (P (TR01872P.1) attP2, THU#2105). Others were UAS-Flag-Rel.68(w (×); P(w(+mC) = UAS-FLAG-Rel.68) i21-B; TM2/TM6C, Sb (1), BDSC# 55778), UAS-BubR1 (P(w(+mC) = UAS-BubR1.Exel)1, y(1) w(1118), BDSC#8385). All genotypes are available in Supplementary Table 1.

### Cell culture and treatments

HeLa, HEK293T, and HepG2 cells were cultured in DMEM supplemented with 10% fetal bovine serum (F101-01, Vazyme, China) at 37°C and 5% CO_2_. Unless specified, starvation conditions consisted of growth in DMEM without FBS for 24 hr.

### Gene silencing using small interfering RNA (siRNA)

In a 24-well plate, reverse transfection was performed on Hela and HepG2 cells at a concentration of 3 × 10^4^ cells/well. Transfection was performed with 20 nM siRNA targeted at hBubR1 or Negative Control siRNA (Youkang, China). Transfection complexes were generated using lipofectamine RNAi MAX in Opti-MEM medium in accordance with guidelines of manufacturer. The ogligoes used in this study were listed as following:

siBUBR1#1-F:AACGGGCAUUUGAAUAUGAAA;
siBUBR1#1-R:UUUCAUAUUCAAAUGCCCGUU;
siBUBR1#2-F:UGCAAGAAGAGACGGAGAA;
siBUBR1#2-R:UUCUCCGUCUCUUCUUGCA.

### Triglyceride measurements

The level of triglyceride (TAG) was quantified using Triglyceride (TG) Content Assay Kit (geruisi, G0910W), following the manufacturer instructions. In brief, 5 beheaded females with indicated phenotypes or treatments were homogenized in 200 ul TG extracts. Homogenized samples were centrifuged at 12000 rpm for 10 min at room temperature, 20 ul of the supernatant extract were used to measure triglycerides, and 10 ul were for quantification of protein concentrations (P0011, Beyotime Biotechnology, China) according to the manufacturer instructions. Normalized TAG levels to protein levels. Note: The kit measures the sum of all kinds of TAGs. 9 replicates for each genotype were carried out. Starvation lasts 72 h in adults and 32 h in larvae of all starvation-associated assays.

### Oil red O staining

Fat body or carcasses of flies with indicated conditions were dissected in PBS, removing all of the eggs and intact intestines, followed with the fixation in 4% paraformaldehyde for 30 min at room temperature, then washed dissected fat bodies twice in PBS, next incubated dissected fat bodies in fresh Oil Red O solution for 30 min (mixture of 6 ml of 0.1% Oil Red O in isopropanol and 4 ml distilled water after passed through a 0.25 mm syringe), finally rinsed the dissected fat body with distilled water twice. Bright-field images of fat bodies were captured via Leica M165 FluoCombi stereoscope system (a single focal plane utilized) and processed using Adobe Photoshop and Leica software. Note: Contrast (red neutral lipids vs. yellow/black cuticle) was enhanced using Adobe Photoshop (equal for all images from the same experiment) in order to better visualize the red stain. Starvation lasts 72 h of all starvation-associated assays.

### Bodipy staining

Fat body/carcasses of flies with corresponding genotypes or treatments were dissected in PBS, removing all of the eggs and intestines. Stained samples for 45 min in a solution which mixed 2 μg/ml Bodipy/LipidTOX (D3922, Thermo Fisher Scientific, USA/2448109, Invitrogen, USA; 1:1000) with 10 μg/ml Hoechst (1:1000) (Hoechst staining is used to locate adipocytes and highlight the differences in lipid droplets on the same horizontal plane), followed rinsing sample with distilled water twice. Then images were captured by Leica confocal system (a single focal plane chose). And quantification of the size (mean area of lipid droplets) of bodipy-stained lipid droplets were carried out via the ImageJ software, analyzed 30 ROI from at least 10 fat bodies per genotype or treatment. Data analysis was performed by using GraphPad Prism 8. Starvation lasts 72 h of all starvation-associated assays.

### Starvation sensitivity analysis/lifespan

7-day-old adult female flies with indicated genotypes (20 flies per vial and 4 vials per cohort) were raised with a filter paper soaking only distilled water (obviously no food), ensuring water in vials was sufficient throughout the whole analysis. The number of dead flies in each cohort was counted every 24 hours, and data is demonstrated as the percent survival of cohorts. At least two independent experiments were conducted.

### RNA-seq

The fat body of adult females with indicated genotypes and treatments were dissected in PBS prepared with DEPC water. Total RNA was extracted of 25–30 female fat body (removing all of intestines and eggs) for RNA-seq. Dissected adult fat body were frozen immediately on liquid nitrogen, followed utilization of isothiocyanate-alcohol phenyl-chloroform for the total RNA preparation. Novogene (China) performed the whole previous sequencing and analysis. The library preparations were sequenced on an Illumina Novaseq platform and 150 bp paired-end reads were generated. Raw data (raw reads) of fastq format were firstly processed through in-house perl scripts, and Q20, Q30 and GC content the clean data were simultaneously calculated. Index of the reference genome was built using Hisat2 v2.0.5 and paired-end clean reads were aligned to the reference genome using Hisat2 v2.0.5. Differential expression analysis of two conditions/groups (two biological replicates per condition) was performed using the DESeq2 R package (1.20.0). Genes with an adjusted *P*-value < 0.05 were assigned as differentially expressed. The local version of the GSEA analysis tool http://www.broadinstitute.org/gsea/index.jsp was utilized for GSEA, and Kyoto Encyclopaedia of Genes and Genomes (KEGG) enrichment was performed by the ClusterProfiler R package. All adult flies were fasted for 72 h.

### RT-qPCR

For qRT-PCR analysis, total RNA was prepared from 25–30 adult flies or dissected fat body with indicated genotypes before and after 72 h-starvation using Animal Total RNA Isolation Kit (FORE GENE, RE-03014), according to the manufacturer’s instruction. Reverse transcription of RNA to cDNA was conducted via using HiScript III RT SuperMix for qPCR (+gDNA wiper) (Vazyme, R323). Quantitative PCR was performed with SYBRGreen (Vazyme, Q711-02) on CFX96TM Real-time PCR System (Bio-Rad, USA). The mRNA expression levels for each gene were normalized to rp49. qPCR primers used in this study were listed as following:

rp49-F: CGTTTACTGCGGCGAGAT;
rp49-R: CCGTTGGGGTTGGTGAG;
DptA-F: GCTGCGCAATCGCTTCTACT;
DptA-R: TGGTGGAGTGGGCTTCATG;
pirk-F: GGCGTTCGTGTGATAG;
pirk-R: CTCAATGCGGTACTCC;
Relish-F: ACAGGACCGCATATCG;
Relish-R: GTGGGGTATTTCCGGC;
Bmm-F: CAATAAGGGTCTGGCCAACTGGAT;
Bmm-R: TAAGTCCTCCACCATTACTCTGGC;
hBubR1-F: CAGCGGCTTTCGGACTGTA;
hBubR1-R: CACAATTCACCATCTTTTAGCTCAG.

### Luciferase assay

Firefly luciferase was measured using Dual Luciferase Reporter Assay Kit (Vazyme, China, DL101-01). The coding sequence of Relish promoter was cloned to PGL3-Basic vector at the KpnI site. ORF sequences of BubR1 and EGFP were cloned to pCDNA3-Flag vector at the EcoRI sites. The construct was transformed into HEK293T cells using Calcium Phosphate Cell Transfection Kit (Beyotime, C0508) for 2 days. Cells were added 100 μl the appropriate amount of 1 × Cell Lysis Buffer provided by the manufacturer into the tube and incubated for 5 min at room temperature. Centrifuge for 2 min, 12000 × g at room temperature, and collect the supernatant for subsequent testing. Add 100 μl of Luciferase Substrate to a 96-well plate. Carefully pipet 20 μl of the sample into the plate. Immediately after mixing rapidly, detect the Firefly luciferase reporter gene activity by full-spectrum microplate reader. Simultaneously, add 100 μl of stop reaction buffer into the plate to detect Renilla luciferase activity. The luciferase activity was determined by Firefly/Renilla luciferase. Three replicates for each genotype and condition were performed.

### Western blotting

To test the protein level of Relish, carcasses of flies with indicated genotypes were dissected in PBS, removing all of the eggs and intact intestines. Dissected adult fat bodies were frozen immediately on liquid nitrogen and incubated in 200 μl TBST. After clarification at 12,000 rpm for 20 min at 4°C and supernatant were used for Western blotting analyses. The antibodies for this assay are anti-Relish (130-10080, Raybiotech, USA), anti-BubR1 (sc-47744, Santa Cruz, USA), anti-Tubulin (2148S, CST, USA).

### Ubiquitination assay

To assay for the effect of BubR1 on the ubiquitination of Relish, carcasses of flies with indicated genotypes were dissected in PBS, removing all of the eggs and intact intestines. Dissected adult fat bodies were frozen immediately on liquid nitrogen and incubated in 200 μl TBST with 10 μM MG132 for 30 min. After clarification at 12,000 rpm for 20 min at 4°C, 20 μl of anti-Flag (Sigma, A2220) bead slurry was added into the supernatant and rotated for 4 h at 4°C, followed by washing the beads with 1 ml of TBST four times each. The beads and supernatant were used for Western blotting analyses for ubiquitin and other proteins. The antibodies for this assay are anti-ubiquitin (3936T, CST, USA), anti-DDDDK (ATVF15021, Abkine, USA), anti-Tubulin (2148S, CST), Goat anti-mouse HRP (A0216, Beyotime).

### Statistical analysis

Statistical analysis was conducted using Prism 8 (GraphPad Software, USA). Except experiments of starvation resistance using Log-rank tests, differences between groups were assessed using unpaired two-tailed Student’s *t*-tests. A value of *p* < 0.05 was considered statistically significant.

### Software and data availability

Prism 8.0 (GraphPad) utilized in the study is available at https://www.graphpad.com/. The Adobe Photoshop CC 2021 and Adobe Illustrator 2020 used are available at https://www.adobe.com/products/catalog.html. The Leica Application Suite X (LAS X) chosen is available at https://www.leica-microsystems.com/products/microscope-software/p/leica-las-x-ls/downloads/. All RNA-seq datasets are publicly available in Sequence Read Archive (SRA) BioProject ID: PRJNA924253 (corresponding to data in Figure 3). Source data for Figure 3B to C have been provided as Supplementary Table 2. Any raw data supporting the finding of this project are obtainable from the corresponding author, based on reasonable request.

## Supplementary Materials

Supplementary Figures

Supplementary Table 1

Supplementary Table 2
