# Supplementary material for: BubR1 controls starvation-induced lipolysis via IMD signaling pathway in Drosophila
Source: Aging (Albany NY). 2024 Feb 8;16(4):3257–79. doi: 10.18632/aging.205533 (PMC10929803; doi:10.18632/aging.205533)
Supplement: Supplementary Figures [file aging-16-205533-s001.pdf]

SUPPLEMENTARY FIGURES

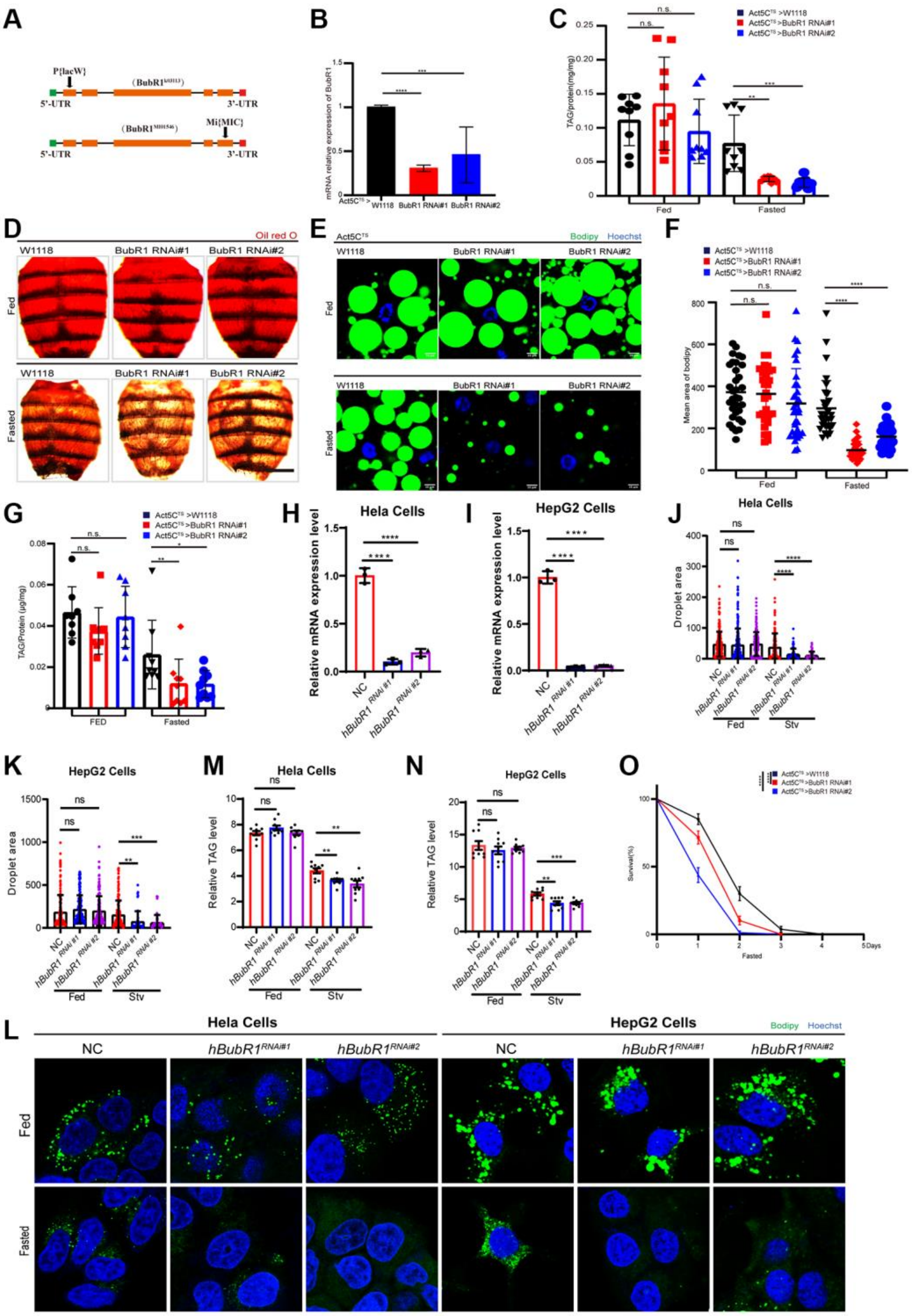

Supplementary Figure 1. Whole-body knockdown of BubR1 promotes lipid metabolism under starvation. Related Figure 1. (**A**) The schematic diagrams of genomic information for BubR1mutants. Homozygous BubR1 mutant flies BubR1MI01546 contains an insertion of transposable Minos (Mi (MIC)) element in the 3′ untranslated region (UTR) of the BubR1 gene, while BubR1k03113 contains a transgenic insertion based on P-element(P(lacW)) in the 5′ untranslated region (UTR) of the BubR1 gene. (**B**) The BubR1 mRNA level of flies with ubiquitous expression of w1118, BubR1 RNAi#1, BubR1 RNAi#2 transgenic lines using the *Act5C-GAL4; tub-gal80ts* driver (named Act5C^{TS}). Results are representative of three biological repetitions (mean ± SD). Unpaired two-tailed *t*-test was performed. $^{***}p < 0.001$, $^{****}p < 0.0001$. (**C**) Total triglyceride (TAG) levels of whole flies with ubiquitously expressing w1118, BubR1 RNAi#1, BubR1 RNAi#2 via Act5C^{TS} before and after starvation (72 h). $n = 9$ samples. Data are presented as mean and SD. Unpaired two-tailed *t*-test was performed. $^{**}p < 0.01$, $^{***}p < 0.001$ and n.s.: non-significant represents $p > 0.05$. (**D**) Representative images of Oil red O (ORO) stain of dissected fat body of flies

with ubiquitous expression of w1118, BubR1 RNAi#1, BubR1 RNAi#2 transgenic lines using the driver Act5C^{TS} before and after starvation (72 h). Scale bars represent 1000 µm. (**E**) Bodipy stain of dissected carcass/fat body of flies with ubiquitous expression of w1118, BubR1 RNAi#1, BubR1 RNAi#2 transgenic lines using Act5C^{TS} before and after starvation (72 h). Bodipy (neutral lipids; green) and Hochest (Hochest; blue) detected by fluorescent histochemistry. Scale bars represent 10 µm. (**F**) Quantification of the mean area of lipid droplets among the more than 30 ROI (region of interest) from flies before and after starvation (72 h) with indicated genotypes. Each dot corresponds to one ROI from indicated genotypes or treatments. Data are presented as mean and SD. Unpaired two-tailed *t*-test was performed. $^{****}p < 0.0001$ and Abbreviation: n.s.: non-significant represents $p > 0.05$. (**G**) Total triglyceride (TAG) levels of larvae with ubiquitously expressing w1118, BubR1 RNAi#1, BubR1 RNAi#2 via Act5C^{TS} before and after starvation (32 h). $n = 9$ samples. Data are presented as mean and SD. Unpaired two-tailed *t*-test was performed. $^{**}p < 0.01$, $^{***}p < 0.001$ and Abbreviation: n.s: non-significant represents $p > 0.05$. (**H**, **I**) Relative mRNA levels of hBubR1 in Hela and HepG2 cells after hBubR1 RNAi. *P*-values were calculated from respective control using an unpaired Student's *t*-test. Results are representative of three biological repetitions (mean ± SEM). $^{****}p <$ 0.0001.(**J**, **K**) The dot graph of the mean area of lipid droplets among the more than 30 ROI (region of interest) from NC, hBubR1 RNAi before and after starvation (24 h). Each dot corresponds to one ROI. (**L**) Bodipy stain of lipid droplet of NC, hBubR1 RNAi before and after starvation (24 h). Bodipy (neutral lipids; green) and Hoechst (Hoechst; blue) detected through fluorescent histochemistry. (**M**, **N**) Quantification of total triglyceride (TAG) levels of cells in control and hBubR1 RNAi before and after starvation (24 h). $n = 9$ samples. (**O**) Starvation resistance of female flies with indicated genotypes. $n = 4$ cohorts (total 80 flies). Data are presented as percents and SE. $n = 4$ cohorts (total 80 flies). Data are presented as percents and SE. $^{****}P < 0.0001$.

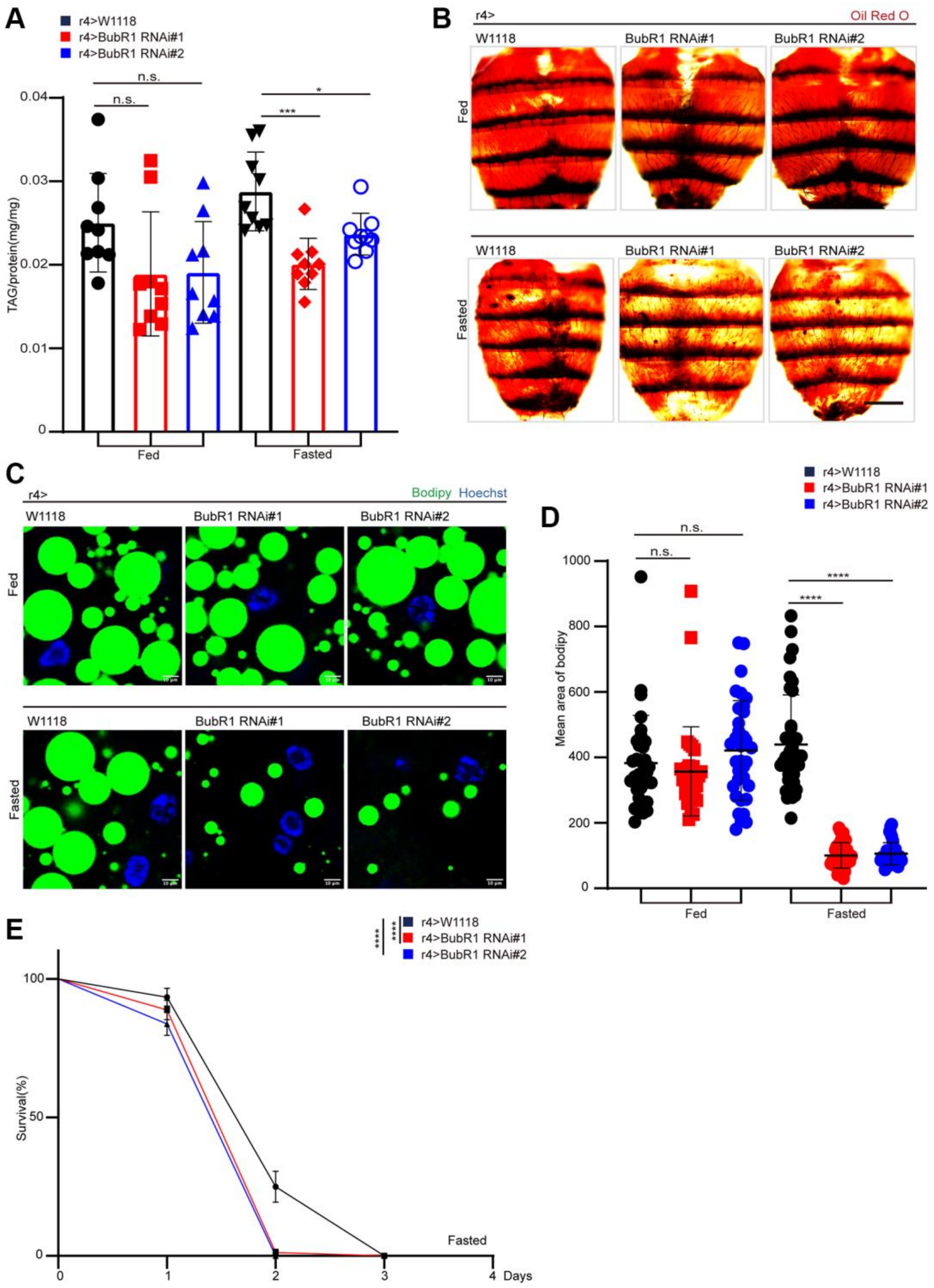

Supplementary Figure 2. Fat body-knockdown of BubR1 controls lipid metabolism in Drosophila upon fasting. Related Figure 2. (**A**) Total triglyceride (TAG) levels of whole flies before and after starvation (72 h) with specially expressing w1118, BubR1 RNAi#1 and BubR1 RNAi#2 in the fat body driven by r4-GAL4. *n* = 9 samples. Results are representative of three biological repetitions (mean ± SD). Unpaired two-tailed *t*-test was performed. $^{*}p < 0.05$, $^{***}p < 0.001$ and Abbreviation: n.s.: non-significant represents $p > 0.05$. (**B**) Oil red O (ORO) stain of dissected fat body with indicated genotypes before and after starvation (72 h). Scale bars represent 1000 µm. (**C**) Bodipy stain of dissected carcass/fat body of indicated genotype flies before and after starvation (72 h). Bodipy (neutral lipids; green) and Hochest (Hochest; blue) detected by fluorescent histochemistry. Scale bars represent 10 µm. (**D**) Quantification of the mean area of lipid droplets among the more than 30 ROI (region of interest) from flies before and after starvation (72 h) with indicated genotypes. Each dot corresponds to one ROI. Unpaired two-tailed *t*-test was performed. Data are representative of three biological repetitions (mean ± SD). $^{****}p < 0.0001$ and Abbreviation: n.s.: non-significant represents $p > 0.05$. (**E**) Survival curve of female flies with indicated genotypes under fast starvation. *n* = 4 cohorts (total 80 flies). Data are presented as percents and SE. $^{****}p < 0.0001$.

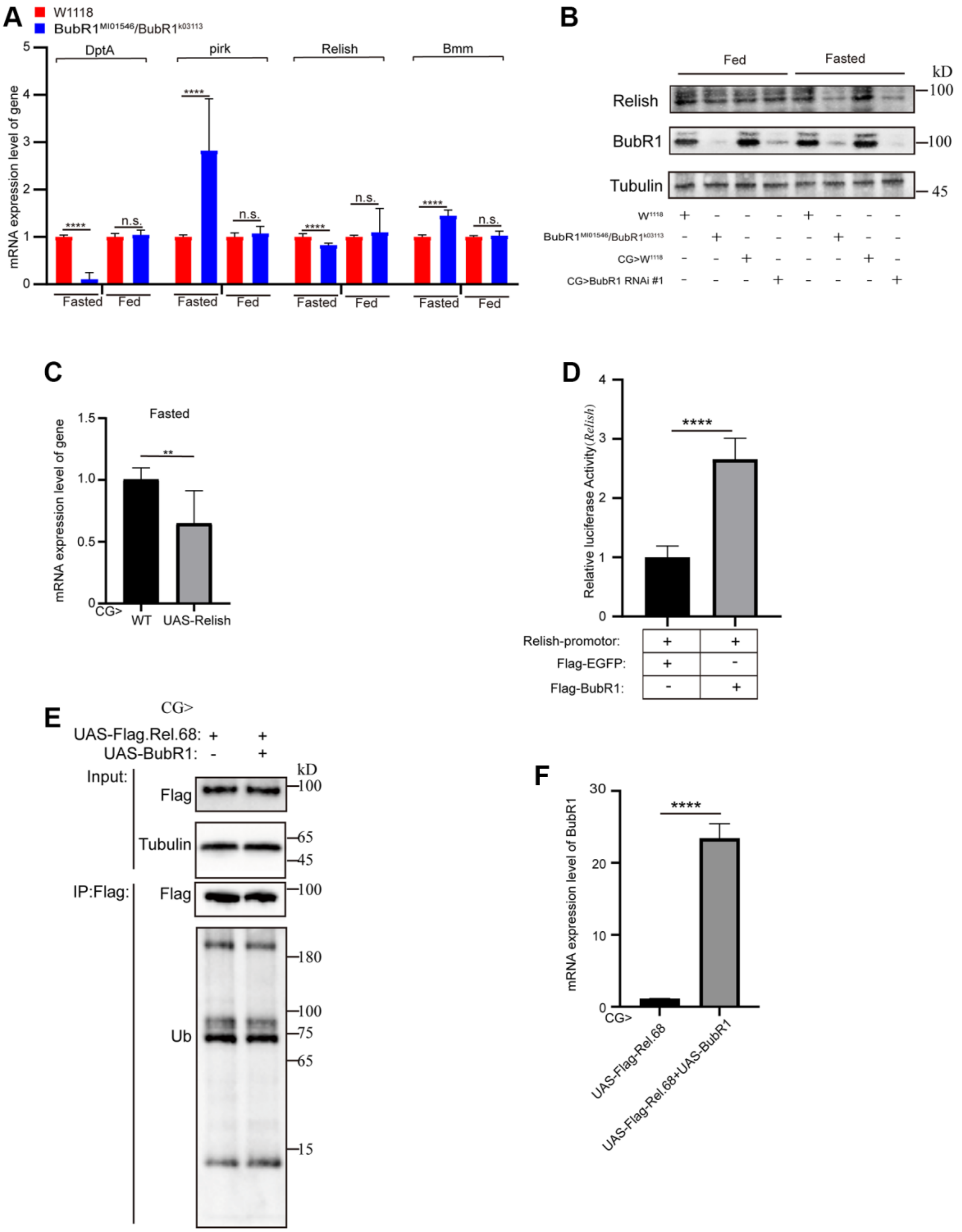

Supplementary Figure 3. BubR1 regulates transcription of Relish during acute starvation. Related Figure 3. (**A**) Relative mRNA levels of DptA, pirk, Relish and Bmm in BubR1 heterozygous mutant flies before and after starvation. *P*-values were calculated from respective control using an unpaired Student's *t*-test. Results are representative of three biological repetitions (mean ± SEM). $^{****}p < 0.0001$. (**B**) The protein level of Relish in fat bodies from flies with indicated genotypes upon fed and fasting (W^{1118}, $BubR1^{MI01546}/BubR1^{K03113}$, CG>$W^{1118}$ and CG>BubR1 RNAi #1). Tubulin, loading control. (**C**) The change of pirk mRNA level in flies with Relish overexpression in fat bodies under starvation. (**D**) Luciferase reporter experiments using a Relish gene promoter (−2542 to 1 bp from the the transcription start site) in HEK293T cells. $n = 3$ biological replicates, error bar represents SD. (**E**) Ubiquitination of Relish in fat bodies from flies with indicated genotypes upon fasting (CG>UAS-Flag-Rel.68, CG>UAS-Flag-Rel.68+UAS-BubR1). Tubulin, loading control. (**F**) The mRNA level of BubR1 in fat bodies from indicated genotypes upon fasting (CG>UAS-Flag-Rel.68, CG>UAS-Flag-Rel.68+UAS-BubR1). Results are representative of three biological repetitions, data were represented as mean ± SEM, Student's *t*-test was used. $^{****}p < 0.0001$.

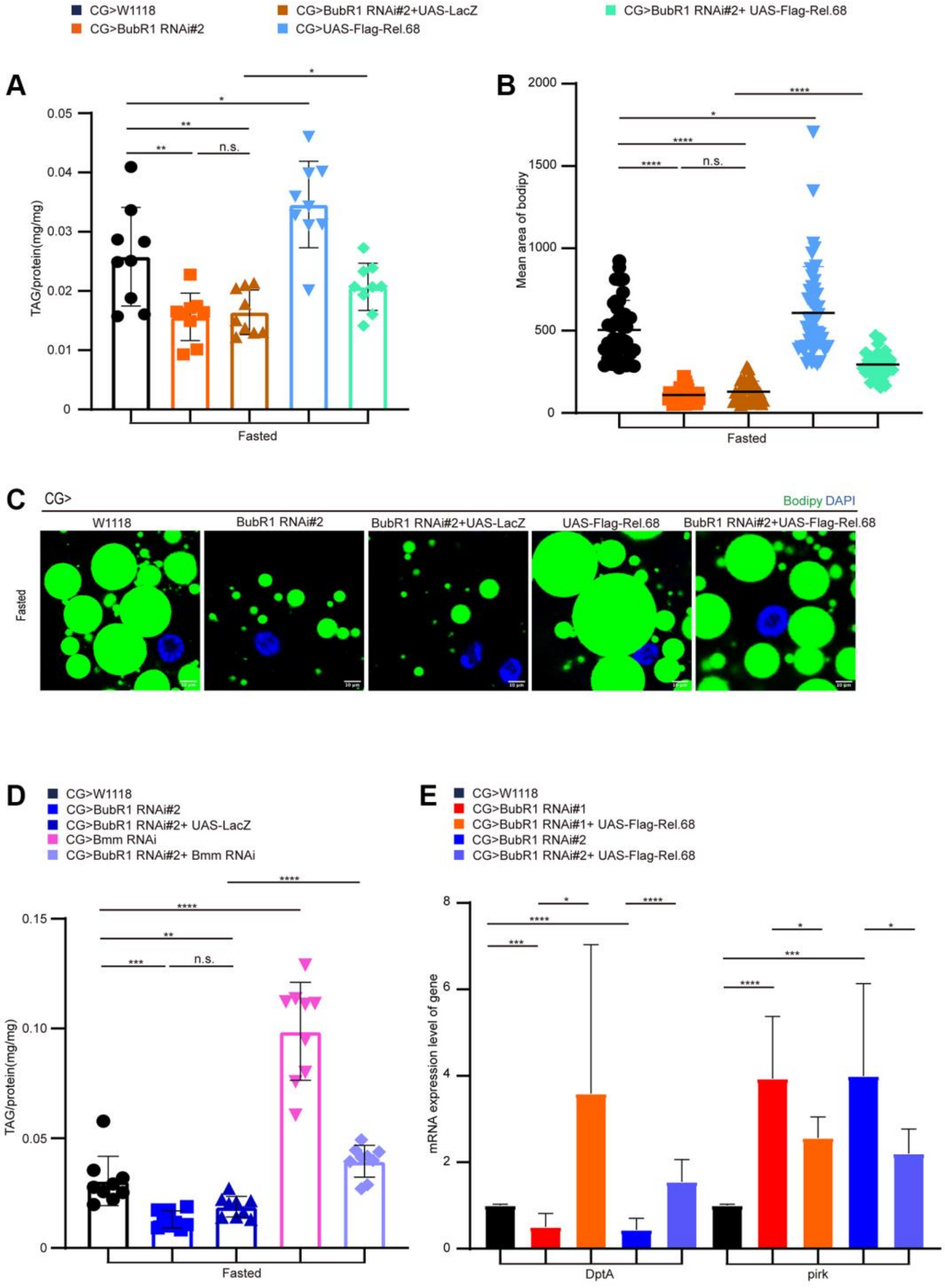

Supplementary Figure 4. BubR1 suppresses lipolysis by inhibiting Relish-mediated Bmm expression upon fasting. Related Figure 4. (**A**) TAG measurement of flies with specially expressing W1118, BubR1 RNAi#2, BubR1 RNAi#2+UAS-LacZ, UAS-Flag-Rel.68, and UAS-Flag-Rel.68 with BubR1 RNAi#2 in the fat body driven by CG-GAL4. n = 9 samples. Data are presented as mean and SD. Unpaired two-tailed t-test was chosen. $^{*}p < 0.05$, $^{**}p < 0.01$. (**B**) Quantification of the mean area of lipid droplets among more than 30 ROI (region of interest) in flies under starvation with corresponding genotypes. Each dot corresponds to one ROI. Data are presented as mean and SD. Unpaired two-tailed t-test was performed. $^{*}p < 0.05$, $^{****}p < 0.0001$. (**C**) Bodipy staining of flies with specially expressing W1118, BubR1 RNAi#2, BubR1 RNAi#2+UAS-LacZ, UAS-Flag-Rel.68, and expressing UAS-Flag-Rel.68 with BubR1 RNAi#2 in the fat body under starvation. Bodipy (neutral lipids; green) and Hochest (Hochest; blue) detected by fluorescent histochemistry. Scale bars represent 10 µm. (**D**) TAG level of flies with specially expressing W1118, BubR1 RNAi#2, BubR1 RNAi#2+UAS-LacZ, and expressing BubR1 RNAi#2 with Bmm RNAi in the fat body driven by CG-GAL4. n = 9 samples. Data are presented as mean and SD. Unpaired two-tailed t-test was chosen. $^{****}p < 0.0001$. (**E**) The DptA and pirk mRNA level of fat bodies from females with expressing W1118, BubR1 RNAi#1, both BubR1 RNAi#1 and UAS-Flag-Rel.68, BubR1 RNAi#2, both BubR1 RNAi#2 and UAS-Flag-Rel.68 in the fat body via CG-GAL4. P-values were calculated from respective control using an unpaired Student's t-test. Results are representative of three biological repetitions (mean ± SD). $^{*}p < 0.05$, $^{***}p < 0.001$, $^{****}p < 0.0001$.
