# Supplementary material for: BubR1 controls starvation-induced lipolysis via IMD signaling pathway in Drosophila
Source: Aging (Albany NY). 2024 Feb 8;16(4):3257–79. doi: 10.18632/aging.205533 (PMC10929803; doi:10.18632/aging.205533)
Supplement: Supplementary Table 1 [file aging-16-205533-s002.pdf]

## SUPPLEMENTARY TABLE

**Supplementary Table 1. Full *Drosophila* genotypes as they appear in each figure panel, related to Figures 1–4 and Supplementary Figures 1–4.**

| | |
|---|---|
| **Figure 1** | |
| **A** | $w^-$; +/+; +/+ |
| | $w^-$; $BubR1^{MI01546}/BubR1^{MI01546}$; +/+ |
| **B, C, D, F** | $w^-$; $BubR1^{MI01546}/BubR1^{MI01546}$; +/+ |
| | $w^-$; +/+; +/+ |
| | $w^-$; $BubR1^{MI01546}/BubR1^{k03113}$; +/+ |
| **E** | $w^-$; +/+; +/+ |
| | $w^-$; $BubR1^{MI01546}/BubR1^{k03113}$; *r4-Gal4* |
| | *UAS-BubR1*; $BubR1^{MI01546}/BubR1^{k03113}$; *r4-Gal4* |
| **Figure 2** | |
| **A** | $w^-$; +/+; +/+ |
| **B, C, D, E, F** | $w^-$; *CG-Gal4*; +/+ |
| | +/+; *CG-Gal4*; *BubR1 RNAi#1* |
| | +/+; *CG-Gal4*; *BubR1 RNAi#2* |
| **Figure 3** | |
| **A, B, C, D, E, F** | $w^-$; *CG-Gal4*; +/+ |
| | +/+; *CG-Gal4*; *BubR1 RNAi#1* |
| **Figure 4** | |
| **A, B, C** | $w^-$; *CG-Gal4*; +/+ |
| | +/+; *CG-Gal4*; *BubR1 RNAi#1* |
| | +/+; *CG-Gal4*; *BubR1 RNAi#1,UAS-LacZ* |
| | +/+; *CG-Gal4*; *BubR1 RNAi#1,UAS-Flag-Rel.68* |
| **D** | $w^-$; *CG-Gal4*; +/+ |
| | +/+; *CG-Gal4*; *BubR1 RNAi#1* |
| | +/+; *CG-Gal4*; *BubR1 RNAi#1,UAS-Flag-Rel.68* |
| | +/+; *CG-Gal4*; *BubR1 RNAi#2* |
| | +/+; *CG-Gal4*; *BubR1 RNAi#2,UAS-Flag-Rel.68* |
| **E** | $w^-$; *CG-Gal4*; +/+ |
| | +/+; *CG-Gal4*; *BubR1 RNAi#1* |
| | +/+; *CG-Gal4*; *BubR1 RNAi#1,UAS-LacZ* |
| | +/+; *CG-Gal4*; *BubR1 RNAi#1, Bmm RNAi* |
| **Supplementary Figure 1** | |
| **B, C, D, E, F, G** | $w^-$; *Act5C-GAL4*; *tub-gal80ts* |
| | +/+; *Act5C-GAL4*; *tub-gal80ts/BubR1 RNAi#1* |
| | +/+; *Act5C-GAL4*; *tub-gal80ts/BubR1 RNAi#2* |
| **Supplementary Figure 2** | |
| **A, B, C, D, E** | *w*; $+/+^-$; *r4-Gal4* |
| | +/+; +/+; *r4-Gal4, BubR1 RNAi#1* |
| | +/+; +/+; *r4-Gal4, BubR1 RNAi#2* |

| Supplementary Figure 3 | |
|---|---|
| **A** | *w⁻; +/+; +/+* |
| | *$w^-$; $BubR1^{MI01546}/BubR1^{k03113}$; +/+* |
| **B** | *$w^-$; CG-Gal4; +/+* |
| | *+/+; CG-Gal4; UAS-Flag-Rel.68* |
| **D, E** | *+/+; CG-Gal4; UAS-Flag-Rel.68* |
| | *$X^{UAS\text{-}BubR1}$; CG-Gal4; UAS-Flag-Rel.68* |

| Supplementary Figure 4 | |
|---|---|
| **A, B, C** | *$w^-$; CG-Gal4; +/+* |
| | *+/+; CG-Gal4; BubR1 RNAi#2* |
| | *+/+; CG-Gal4; BubR1 RNAi#2,UAS-LacZ* |
| | *+/+; CG-Gal4; BubR1 RNAi#2,UAS-Flag-Rel.68* |
| **D** | *$w^-$; CG-Gal4; +/+* |
| | *+/+; CG-Gal4; BubR1 RNAi#2* |
| | *+/+; CG-Gal4; BubR1 RNAi#2,UAS-LacZ* |
| | *+/+; CG-Gal4; BubR1 RNAi#2, Bmm RNAi* |
| **E** | *$w^-$; CG-Gal4; +/+* |
| | *+/+; CG-Gal4; BubR1 RNAi#1* |
| | *+/+; CG-Gal4; BubR1 RNAi#1,UAS-Flag-Rel.68* |
| | *+/+; CG-Gal4; BubR1 RNAi#2* |
| | *+/+; CG-Gal4; BubR1 RNAi#2,UAS-Flag-Rel.68* |
